# The neural representation of competence traits: An fMRI study

**DOI:** 10.1038/srep39609

**Published:** 2016-12-20

**Authors:** Ning Ma, Simin Wang, Quansen Yang, Tingyong Feng, Frank Van Overwalle

**Affiliations:** 1 School of Psychology, Center for Studies of Psychological Application, Guangdong Key Laboratory of Mental Health & Cognitive Science, South China Normal University, Guangzhou, China; 2 School of Psychology, Southeast University, Chongqing, China; 3 Department of Psychology, Vrije Universiteit Brussel, Brussels, Belgium

## Abstract

Previous neuroimaging studies have revealed that a trait code is mainly represented in the ventral medial prefrontal cortex (vmPFC). However, those studies only investigated the neural code of warmth traits. According to the ‘Big Two’ model of impression formation, competence traits are the other major dimension when we judge others. The current study explored the neural representation of competence traits by using an fMRI repetition suppression paradigm, which is a rapid reduction of neuronal responses upon repeated presentation of the same implied trait. Participants had to infer an agent’s trait from brief behavioral descriptions that implied a competence trait. In each trial, the critical target sentence was preceded by a prime sentence that implied the same or opposite competence-related trait, or no trait. The results revealed robust repetition suppression from prime to target in the vmPFC and precuneus during trait conditions. Critically, the suppression effect was much stronger after being primed with a similar and opposite competence trait compared with a trait-irrelevant prime. This suppression pattern was found nowhere else in the brain. Consistent with previous fMRI studies, we suggest that the neural code of competence traits is represented in these two brain areas with different levels of abstraction.

Forming impressions of other people is one of the fundamental tasks of human social cognition. A well-established view in social cognition is that people form impressions of others on two fundamental trait dimensions: warmth and competence^1^,^2^,^3^, also called the “Big Two” dimensions of social impression formation^4^. The warmth dimension captures traits that are related to the perceived quality of social relationships, including friendliness, helpfulness, sincerity, trustworthiness and morality, whereas the competence dimension reflects traits that are related to perceived ability, including intelligence, skill, creativity and efficacy. While warmth traits reflect the quality of our social relationships, competence traits provide distinct information that is important for social collaboration and survival. This dimension will prevail when we select partners to cooperate on tasks at work or leisure time such as sports (where competence is critical for success) or for long-term sexual relationships (where competence is required to provide offspring with health, food and shelter). Warmth fulfils our basic need to belong, while competence fulfils our need to successfully reaching our goals.

Recent neuroimaging research has revealed that forming impressions of others recruits a network of brain areas, named the mentalizing network^5^,^6^,^7^. It was suggested that in this mentalizing network, the medial prefrontal cortex (mPFC) is a key area that integrates social information at a more abstract level, such as the agent’s traits^5^. The mPFC is typically divided into at least two sub-regions, the dorsal mPFC and ventral mPFC. The dorsal mPFC has been associated with mentalizing about people that are dissimilar from oneself, while the ventral aspect has been linked to mentalizing about persons perceived to be similar to the self^5^,^8^. The aim of the present research is to investigate whether and how trait knowledge related to an agent’s competence is encoded and represented in the brain?

The idea that knowledge and memories are represented in neural representations or codes was proposed by Wood and Grafman^9^ (2003) who described representations as distributed memories in the brain that encode information and, when activated, facilitate access to and integration with the stored information. This idea was further developed in a structured event complex framework in which the mPFC represents integrative information that gives rise to social attitudes and knowledge, including traits. If a trait code exists, this facilitates processing of trait-related information, since such a code may immediately render accessible not only prior social knowledge about traits, but also related knowledge about behaviors, intentions and potential responses. For instance, the trait “aggressive” immediately brings to mind typical behaviors (e.g., giving a slap), the underlying intention (e.g., intentional hurting), and potential emotional responses (e.g., fear or anger^10^). Such knowledge may allow us to recognize behaviors immediately as revealing an underlying trait of the agent.

To localize a trait code, previous neuroimaging studies have applied fMRI repetition suppression^11^,^12^,^13^. Repetition suppression refers to the observation that repeated presentations of a stimulus or concept consistently reduces fMRI responses relative to the presentations of a novel stimulus or concept^14^,^15^,^16^. Although there are many possible theoretical explanations, fMRI repetition suppression has generally been taken as evidence for a neural representation that reveals the invariant features of stimuli or concepts, whereas recovery from repetition implies selectivity of the neural population to a specific stimulus or concept^15^. Prior research on trait judgments has demonstrated that traits inferred from different behavioral descriptions involving different agents that have little semantic or conceptual associations except for the implied trait, lead to a decrease in brain activation in the ventral part of the mPFC^11^,^12^,^13^,^17^. Thus, the ventral mPFC may house a trait code abstracting out the shared trait implication from varying lower-level behavioral information.

However, most fMRI studies of trait representation using repetition suppression focused on warmth traits, but neglected competence-related traits. Nevertheless, as noted earlier, when we judge others, we often describe them in terms of two fundamental content dimensions: warmth or sociality and competence. In a recent fMRI repetition suppression study^18^, the authors explored how different warmth and competence traits reflecting the “Big Two” are represented as trait codes in the brain. In their research, a warmth trait-implying target description (e.g., implying nice) was preceded by a prime description that implied the same trait, or a dissimilar competence trait which also differed in valence (e.g., unintelligent). The unexpected finding of this study was that suppression was revealed even when a competence-implying prime sentence was followed by a different warmth-implying target sentence. These results were explained as indicating that people categorize a combination of competence and warmth information into novel trait subcategories, reflecting either nice (but incompetent) traits or nerdy (but socially awkward) traits. However, there were several limitations in this study that may shed doubt on these conclusions. First of all, the design included a low number of competence traits in comparison with warmth traits. Second and perhaps more importantly, the study investigated only the combination of warmth and competence traits (with opposite valences), but failed to uncover the neural code of competence traits alone.

Therefore, in the current study, we focused solely on the dimension of competence and explored the neural representation of competence traits by using fMRI repetition suppression. It is possible that competence is not encoded in the same manner as warmth traits. First, competence does not refer directly to social interaction while most warmth traits do (friendly, trustworthy, honest, etc). The judgments of warmth carry more weight in social interaction and precede competence judgments, because good or bad intentions of another person are very crucial to survival^2^,^19^. Second, competence often refers to performance on specific tasks that are likely to differ more in content, such as intellectual, artistic, and athletic domains of competence. Compared to warmth traits associated with the valence of a social judgment, competence traits are more related with the extremity of an impression^20^. As such, competence traits might be more concrete and variable than warmth traits, leading perhaps to less abstract encoding in the brain. To explore competence codes, in analogy with previous research on warmth trait codes^11^,^12^, we presented a behavioral description (prime sentence) followed by another behavioral description (target sentence) implying a competence trait. We created three conditions by preceding the target description (e.g. implying intelligent) by a prime description that implied the same trait (e.g. intelligent), implied the opposite trait (e.g. unintelligent), or implied no trait at all (i.e. trait-irrelevant). Prior work on warmth traits^11^,^12^,^18^ revealed that the brain encodes opposing traits as belonging to the same trait concept (e.g., including low to high friendliness). Our goal was to compare the pattern of repetition for competence traits in close analogy with previous studies of warmth traits^11^,^12^ by using exactly the same design. That is, we want to investigate whether the same and opposite competence traits (e.g., including low to high intelligence) show a similar suppression effect as was also the case for warmth traits. We predict stronger repetition suppression in the mPFC when the two behavioral description imply the same concept of a competence trait (i.e., similar or opposite competence), and a much weaker repetition suppression effect when the prime sentence is trait-irrelevant.

## Method

### Participants

There were 21right-handed participants (11 women), with ages varying between 19 and 26 years old. Four additional participants were excluded, because of excessive movement artifacts. In exchange for their participation, they were paid 50 RMB (approximately 8 dollars). Participants reported no abnormal neurological history and had normal or corrected-to-normal vision. All participants signed an informed consent form before the experiment. The experiment was conducted in accordance with the Declaration of Helsinki (BMJ 1991; 302: 1194) and was approved by the Medical Ethical Committee of the South China Normal University (of the principal investigator N.M.).

### Procedure and stimulus material

We created three conditions: similar, opposite, and irrelevant. Participants read two sentences concerning different agents who were engaged in behaviors that implied positive or negative competence traits. The target sentence (e.g. ‘Tolvan ranked first in the Physics exam’ to induce the trait competent) was preceded by a prime sentence that implied the same trait (Similar condition, e.g. ‘Calpo got an A from his teacher’), the opposite trait (Opposite condition, e.g. ‘Fatys got fired twice in a week’), or no trait at all (Irrelevant condition, e.g. ‘Wepis felt a quite fresh breeze’). After each trial of two sentences, participants were instructed to infer the agent’s trait from the last (target) sentence and indicated by pressing a button whether a given trait applied to the target description. The trait displayed was either the implied trait or its opposite, so that half of the correct responses were ‘yes’, and the other half were ‘no’. To avoid the possibility that participants would ignore the (first) prime sentence and would only pay attention to the target sentence, we also added a Singleton condition consisting of a single trait-implying behavioral sentence, immediately followed by a trait question. Hence, during the first sentence of any trial, the participants could not predict whether a question would or would not appear afterwards, and this ensured carefully reading of all sentences by the participants. There were 20 trials in each condition. Positive or negative trait sentences were counterbalanced between conditions, so that each set of prime and target sentences was used in different conditions for different participants.

The sentences were originally borrowed from earlier studies on competence traits^3^. We translated the material into Chinese and adapted the sentences for a Chinese context and culture. Before the fMRI study, a pilot study (*n* = 210) was conducted to test the experimental stimuli. We only selected the behavioral sentences when at least 90% of the participants agreed that the sentences described competence traits (in a positive or negative manner). Among the selected sentences, there was no significant difference on valence between the positive and negative behavioral sentences and neither between the prime and target trait-related sentences. All the Chinese sentences were presented to the participants and consisted of 11 words (except 11 sentences with 10 words). To avoid associations with a familiar and/or existing name, fictitious ‘Trek’-like names were used^10^,^21^,^22^. To exclude any possible suppression effect resulting from the agent, the agents’ names differed in all the behavioral sentences. All the sentences were presented one at the time in the middle of the screen for a duration of 4 s. To optimize the estimation of the event-related fMRI response, each prime and target sentence was separated by a variable interstimulus interval of 2.5 to 4.5 s randomly drawn from a uniform distribution, during which participants passively viewed a fixation crosshair. After each trial, a fixation cross was shown for 500 ms and then the trait question appeared until a response was given. We created four versions of the material by counterbalancing the trait-relevant sentences of the target sentences among all the three conditions and Singleton condition. We presented one of four versions of the material, counterbalanced between conditions and participants.

### Imaging Procedure

Images were collected with a 3 Tesla Magnetom Trio MRI scanner system (Siemens medical Systems, Erlangen, Germany), using a12-channel radiofrequency head coil. Stimuli were projected onto a screen at the end of the magnet bore that participants viewed by way of a mirror mounted on the head coil. Stimulus presentation was controlled by E-Prime 2.0 (www.pstnet.com/eprime; Psychology Software Tools) under Windows XP. Immediately prior to the experiment, participants completed a brief practice session. Foam cushions were placed within the head coil to minimize head movements. We first collected a high-resolution T1-weighted structural scan (MP-RAGE) followed by 4 functional runs (30 axial slices; 4 mm thick; 1 mm skip). Each run lasted 8 minutes. Functional scanning used a gradient-echo echo planar pulse sequence (TR = 2 s; TE = 33 ms; 3.5 × 3.5 × 4.0 mm in-plane resolution).

### Image Processing and Statistical Analysis

The fMRI data were preprocessed using SPM8 and analyzed using SPM12 (Wellcome Department of Cognitive Neurology, London, UK). For each functional run, data were preprocessed to remove sources of noise and artifacts. Functional data were corrected for differences in acquisition time between slices for each whole-brain volume, realigned within and across runs to correct for head movement, and coregistered with each participant’s anatomical data. Functional data were then transformed into a standard anatomical space (2 mm isotropic voxels) based on the ICBM 152 brain template (Montreal Neurological Institute). Normalized data were then spatially smoothed (8 mm full-width-at-half-maximum [FWHM]) using a Gaussian kernel. Afterwards, realigned data were examined, using the Artifact Detection Tool software package (ART; http://web.mit.edu/swg/art/art.pdf; http://www.nitrc.org/projects/artifact_detect), for excessive motion artifacts and for correlations between motion and experimental design, and between global mean signal and the experimental design. Outliers where identified in temporal difference series by assessing between-scan differences (Z-threshold: 3.0, scan to scan movement threshold 0.45 mm; rotation threshold: 0.02 radians). These outliers were omitted in the analysis by including a single regressor for each outlier (bad scan). No correlations between motion and experimental design or global signal and experimental design were identified.

Next, single participant (1st level) analyses were conducted. Statistical analyses were performed using the general linear model of SPM12 of which the event-related design was modeled with one regressor for each condition, time-locked at the presentation of the prime and target sentences and convolved with a canonical hemodynamic response function with an event duration of 0. Six motion parameters from the realignment as well as outlier time points (identified by ART) were included as nuisance regressors. The response of the participants was not modeled. We used a default high-pass filter of 128 seconds and serial correlations were accounted for by the default autoregressive AR(1) model.

For the group (2nd level) analyses, all clusters were thresholded at an initial voxel-level of an uncorrected *p* < 0.001, and next we considered only clusters that surpassed a cluster-level threshold corrected at a family-wise error (FWE) of *p* < 0.05. We defined suppression as the contrast (i.e. decrease in activation) between prime and target sentence. This suppression contrast was further analyzed in a conjunction analysis (combining all trait conditions) to identify the brain areas commonly involved in the trait inference process. More critically, in line with earlier repetition research^11^,^12^,^13^,^18^, we computed an interaction analysis (with a Similar + Opposite > Irrelevant contrast, with contrast weights 1 −1 1 −1 −2 2, which refer to the Prime and Target respectively in the Similar/Opposite/Irrelevant conditions respectively) to isolate the brain areas involved in a trait code^23^.

However, such an interaction analysis might provide too much false positives because the interaction might sometimes become significant if only one contrast of the interaction is significant, while the other is not. To safeguard against such false positives, we used the obtained peak coordinates to define ROIs and then calculated for each ROI the percentage signal change to verify whether the obtained repetition suppression data showed the full expected pattern. This was done in two steps. First, we identified a region of interest (ROI) as a sphere of 4 mm around the peak coordinates from the whole-brain interaction as described earlier. Second, we extracted the percentage signal change in this ROI from each participant using the MarsBar toolbox (http://marsbar.sourceforge.net). We also calculated a suppression index as the percentage signal change of prime minus target condition. These data were analyzed using a t-test with a threshold of *p* < 0.05. Given that the t-test is calculated independently from the whole brain analysis, this provides further validation for the obtained suppression effect.

## Results

### Behavioral results

A repeated-measure analysis of variance was conducted on the reaction times and accuracy rates from the four conditions (Table 1). The timing data revealed a significant effect of trait condition, *F* (1, 20) = 15.79, *p* < 0.001. Participants responded almost equally fast in the three traits conditions, and much faster than the Singleton condition. The accuracy rate data revealed significant differences among conditions, *F* (1, 20) = 6.96, *p* < 0.001. Participants responded with higher accuracy in the Similar and Singleton conditions as compared with the Opposite and Irrelevant condition. Overall, however, accuracy was very high and above 95% in all conditions. Therefore, all trials were included in the fMRI analysis.

### fMRI results – Whole brain analysis

Our analytic strategy for detecting a repetition suppression effect during trait processing was as follows. As a first step, we conducted a whole-brain, random-effects analysis contrasting prime > target trials in the Similar, Opposite and Irrelevant conditions. This was followed by a Similar + Opposite > Irrelevant interaction to isolate the trait code while controlling for potential suppression due to irrelevant information. We also conducted conjunction analyses combining several conditions to identify a common inference process for competence traits.

The whole-brain analysis of the prime > target contrast revealed significant suppression effects (*p* < 0.05, FWE cluster corrected) in the mPFC (Table 2). This suppression effect was observed in all three experimental (Similar, Opposite and Irrelevant) conditions, and also in a conjunction analysis across two and three of these conditions. The finding that suppression was even found under the irrelevant trait condition is consistent with the idea that some minimal amount of a trait inference process takes place given the explicit instructions to infer a trait. No other areas showed suppression effects in one or more experimental conditions.

To identify the brain areas involved in the trait code and to control for some small degree of suppression after irrelevant primes, we conducted a whole-brain interaction analysis of the prime > target suppression effect in which the Similar and Opposite conditions were contrasted against the Irrelevant condition (Table 2). This interaction revealed significant activation in the ventral mPFC and precuneus.

### fMRI results – Regions of interest

**Figure 1:**
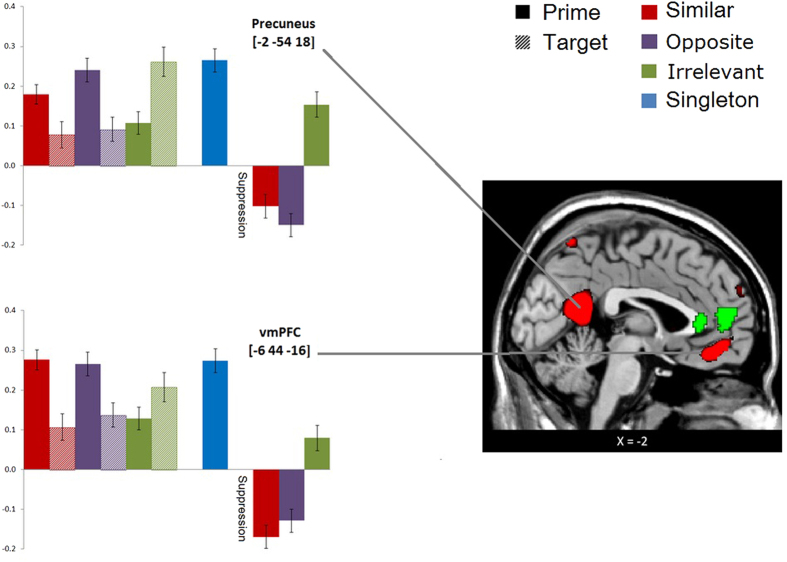
Percent signal change for the prime and target sentences in all conditions, and the repetition suppression index (target - prime condition) based on the ROIs (with MNI coordinates vmPFC: −6, 44, −16; Precuneus: −2, −54, 18). The inset depicts the whole-brain interaction reflecting the trait code (red) and the whole-brain conjunction of the prime > target contrast across all conditions reflecting a common trait inference process given trait-relevant and trait-irrelevant behavioral information (green) with *p* < 0.001 uncorrected.

As a second step in our analysis, we verified that the areas in the whole brain interaction showed the hypothesized repetition suppression pattern for the competence code. Indeed, it is possible that “false” interactions come from differences that do not reflect a trait code, such as differences in prime sentences. To verify that the mPFC and precuneus reveal the predicted repetition suppression effect and, more crucially, that this suppression effect is largest for trait diagnostic as opposed to irrelevant information, we computed activation in two ROIs centered at the whole-brain interaction (with MNI coordinates, vmPFC: −6 44 −16; Precuneus: −2 −54 18). Next, we calculated a repetition suppression index for each ROI by subtracting the percentage signal change in the target sentence from the prime sentence (Fig. 1). The repetition suppression index in the vmPFC clearly showed the predicted pattern: the strongest repetition suppression was found in the Similar condition, becoming non-significantly weaker in the Opposite condition and significantly weaker in the Irrelevant condition. Post hoc two-sided t-tests revealed very strong repetition suppression of the Similar and Opposite conditions in comparison with the Irrelevant condition (*ps* < 0.001). There was no difference between the Similar and Opposite conditions (*p* > 0.22). The repetition suppression index in the precuneus also showed a similar pattern, with a strong repetition suppression effect in the Similar and Opposite conditions compared with the Irrelevant condition (*ps* < 0.001), and no difference between the Similar and Opposite conditions (*p* > 0.23).

To ensure that the mPFC and precuneus were involved only in repetition suppression (i.e. decrease of activation), we also conducted a whole-brain analysis of the reverse target > prime contrast in the Similar, Opposite and Irrelevant conditions. The results revealed a series of brain areas that were more strongly recruited during the presence of the target sentence among the three conditions, including the bilateral thalamus, supplementary motor area (SMA), left inferior parietal gyrus, left lingual gyrus, right insula and right fusiform (Table 3). Importantly, there was no significant mPFC and precuneus activation.

## Discussion

Forming impressions of others is a common but important task in people’s social life. According to the “Big Two” theory, “perceived warmth and competence are the two universal dimensions of human social cognition”^2^. Previous neuroimaging studies have demonstrated that the mPFC is an essential area for inferring traits of others or the self^6^,^10^,^22^. As revealed in earlier fMRI repetition suppression studies, the neural code of warmth traits is represented in the ventral part of mPFC^11^,^12^,^13^. However, very little evidence was documented on the neural code of competence traits. A very recent study^18^ indicated that competence and warmth trait information may be interpreted in terms of novel subcategories, involving combinations of warmth and competence traits (e.g., ‘nice’ and ‘nerdy’). However, due to limitations of the design, this study could not identify the neural representation of competence traits alone in the brain.

The key question of interest in the current study was to identify the neural code of competence traits in the brain by using fMRI repetition suppression. In our study, participants inferred competence traits of others while reading behavioral target sentences that strongly implied a competence trait, after they had read sentences that involved the same competence trait (e.g., intelligent), an opposite competence trait (e.g., unintelligent) or trait-irrelevant information. The results revealed repetition suppression in the ventral mPFC, not only when the prime implied a similar competence trait, but also when it implied an opposite competence trait. These findings were further confirmed by the suppression index, which showed a great amount of repetition suppression for similar and opposite competence traits, but not for trait-irrelevant information, revealing the same pattern of neural suppression of warmth traits^11^,^12^. That opposite competence traits showed the same suppression as similar traits seems to suggest that the vmPFC represents traits in an “absolute” fashion, only reflecting the concept of the trait, regardless of whether they are presented in positive (e.g., intelligent) or negative (i.e., unintelligent) behavior. The same repetition suppression pattern was also found in the precuneus, but not in other brain areas. Note, however, that the precuneus was only identified when keeping constant the irrelevant condition, and not in simple prime > target contrasts. Therefore, the robustness of the precuneus as part of a competence trait code needs to be confirmed in future research. Crucially, the mPFC and precuneus did not show any reverse, enhancement effect. Together, the present findings further extend the representational view of trait codes revealed in previous research^11^,^12^,^18^ by showing that the mPFC represents not only warmth traits, but also competence traits, together making up the “Big Two” dimensions of impression formation.

Our findings on a trait representation in the vmPFC are in line with the idea that the ventral part of mPFC is an anchor of knowledge for mentalizing about other persons. It has been argued that a representation of self-referential stimuli is applied as proxy to ‘simulate’ or ‘project’ our own traits for judging others^8^,^24^,^25^. A recent neuroimaging study revealed that the self is recruited in the ventral mPFC as anchor to evaluate others that are similar to the self, while the dorsal mPFC computes adjustments away from the self while evaluating distant others^26^. This distinction is consistent with other findings showing a ventral-dorsal gradient in the mPFC in value representations of self versus others^27^,^28^. However, the present and prior^11^,^12^ suppression data suggest a subtle difference, in that trait suppression was found in the vmPFC while judging others. This implies that trait knowledge itself is the critical representation in the vmPFC. Although we might use most often traits linked to the self as anchor to judge others, this does not exclude the possibility that we might use traits of others as well. For instance, the use of other-referential traits is more likely when we see ourselves as low on some trait (e.g., unartistic) while recognizing others (e.g., a famous musician) as ideal exemplars for this competence domain.

This study also revealed that the precuneus is involved in repetition suppression of competence traits. Although not always found in repetition suppression research on warmth traits, the precuneus was revealed in one repetition study^12^. Perhaps, the precuneus serves a role in trait representation at a more concrete/indirect level. Prior meta-analyses indicated that the precuneus is involved in mentalizing^29^,^30^,^31^, and its main function might be the construction of a situational scene or context, which includes the integration of relevant behavioral information into a coherent spatial context^32^,^33^, and the retrieval of episodic context information including autobiographic memory^34^. As an important node of the mentalizing network, the precuneus may reflect episodic information, such as a scene or situational background associated with warmth or competent behavior, linked to the vmPFC, the other node in the mentalizing network representing abstract trait concepts. Future studies should investigate the potential connectivity between the precuneus and vmPFC during trait judgments of others.

Alternatively, one may view the current repetition suppression pattern as revealing repetition of the valence implicated by the behavior. It is always the case that similar target traits are similar in valence to the prime, and that opposite target traits are opposite in valence. This suggests that the present repetition suppression effect in the ventral mPFC might be related to evaluative processing when people make competence inferences, rather than the content of inferred traits per se. A series of neuroimaging studies have revealed that the ventral mPFC is recruited during the regulation of emotional processing^35^,^36^,^37^,^38^ and affective mentalizing^39^. However, this interpretation is unlikely. Since repetition suppression did not differ between similar and opposite traits, an interpretation in terms of a valence judgment or an evaluation process is unlikely. Another, related interpretation is that our suppression effect reflects a valence judgment, regardless of whether it is the same or the opposite valence. This interpretation is also unlikely. A previous fMRI study found dissociation between the neural representation of warmth traits and valence^12^. In particular, repetition suppression of positive or negative traits of persons did not show generalization to positive or negative characteristics respectively of objects. This indicates that a trait code in the vmPFC does not carry a generalized valence code, but is limited to the implied trait inference.

The location of the code for competence traits (with MNI coordinates: −6 44 −16) is very similar to the code for warmth traits^11^ (with MNI coordinates: −6 42 −14). This may support the view that the “Big Two” warmth and competence dimensions of person impression formation are represented in the same vmPFC area, although we should be cautious about this observation because the participants differed between these two studies. However, an unresolved issue is whether this area houses specific representations of distinct warmth and competence traits or represents any trait-relevant information. The current design does not provide a satisfactory answer to this question, because it did not provide a direct comparison between warmth and competence traits within the same design. Consequently the data cannot reveal whether these two trait dimensions are reflected by distinct codes that are represented in a distributed manner across the same brain area, or whether these two dimensions are simply part of one common integrative trait code. To answer this question, the overlap in the representation of warmth and competence traits in the vmPFC should be explored. In the previous study by Van Overwalle *et al*. (2015)^18^, the overlap between warmth and competence traits was investigated with dissimilar valence (e.g., nice and nerdy; having a positive and negative connotation respectively), but not with the same valence (e.g., nice and smart, having both a positive connotation). To avoid this limitation, a full factorial design with warmth and competence traits that carry both the same and opposite valence is required. Moreover, a multi-voxel pattern analysis can be applied to test whether warmth and competence traits are represented by similar or different activation patterns distributed across the same brain area.

## Conclusion

The present repetition suppression paradigm offered strong evidence for the representation of competence traits in the ventral mPFC, over and above its role in the processing of trait information. This finding extends the role of vmPFC as an important hub in the representation of both warmth and competence traits, which makes up the “Big Two” dimensions of impression formation.

## Additional Information

**How to cite this article**: Ma, N. *et al*. The neural representation of competence traits: An fMRI study. *Sci. Rep.* **6**, 39609; doi: 10.1038/srep39609 (2016).

**Publisher's note:** Springer Nature remains neutral with regard to jurisdictional claims in published maps and institutional affiliations.

## Figures and Tables

**Table 1:** Response time and accuracy.

Condition	Similar	Opposite	Irrelevant	Singleton
Response Time (ms)	1339_a_	1344_a_	1355_a_	1507_b_
Accuracy rate (%)	98.75_a_	95.83_b_	96.21_b_	98.32_a_

Means in a row sharing the same subscript do not differ significantly from each other according to a Fisher LSD test, *p* < 0.05.

**Table 2:** Repetition Suppression (Prime > Target) of competence traits from the whole brain analysis.

Contrasts and Anatomical Labels	x	y	z	voxels	t
*Similar Traits*					
mPFC	6	56	22	2113	4.68***
	2	30	4		4.18
*Opposite Traits*					
mPFC	−2	32	2	2378	5.55***
	−2	58	16		5.52
*Trait- Irrelevant*					
mPFC	6	48	2	1165	4.74***
	4	34	10		4.52
*Conjunction for Similar and Opposite Traits*					
mPFC	2	54	16	1599	4.61***
	2	30	4		4.32
*Conjunction for Similar, Opposite and Irrelevant Traits*					
mPFC	2	32	4	699	4.24***
	2	52	10		4.24
*Competence suppression:*					
*Interaction of Prime* *>* *Target by Similar* + *Opposite* *>* *Irrelevant [1 −1 1 −1 −2 2]*					
ventral mPFC	−6	44	−16	383	4.89*
	0	36	−18		4.83
Precuneus	−2	−54	18	594	4.93**

Coordinates refer to the MNI (Montreal Neurological Institute) stereotaxic space. Listed are clusters thresholded at *p* < 0.05, FWE cluster-corrected. The contrast weights between straight parentheses refer to the Prime and Target in the Similar/Opposite/Irrelevant conditions respectively. mPFC = medial prefrontal cortex. *p < 0.05, **p < 0.01, ***p < 0.001 (FWE cluster-corrected).

**Table 3:** Repetition Enhancement (Target > Prime) of competence traits from the whole brain analysis.

Contrasts and Anatomical Labels	x	y	z	voxels	t
*Similar Traits*					
R insula	32	22	0	22032	8.04***
L insula	−30	20	0		7.6
SMA	4	16	52		7.55
L Lingual	−10	−74	−6	3285	5.38***
R Fusiform	30	−72	−8		5.09
R Thalamus	12	−18	8	478	5.1**
L Thalamus	−12	−20	6	372	5.04*
*Opposite Traits*					
R insula	32	22	0	52002	9.94***
SMA	2	16	54		9.75
R Superior Temporal	46	−26	−6	1152	5.18***
	52	−40	12		4.61
R Mid-Frontal	30	58	0	776	5.13**
	26	50	10		4.51
Cingulate	−4	−22	28	346	5*
	10	−32	26		4.74
*Trait-Irrelevant*					
SMA	−6	12	54	41274	8.84***
L Superior Parietal	−26	−56	48		8.22
L Inferior Parietal	−28	−60	38		7.87
R insula	32	24	0	679	7.62**
R Thalamus	12	−16	2	1884	6.67***
L Thalamus	−12	−22	6		6.04
Cingulate	−6	−22	28	407	5.11*
*Conjunction of Similar and Opposite Traits*					
R insula	32	22	0	20987	8.04***
L insula	−30	20	0		7.60
SMA	4	16	52		7.55
L Lingual	−20	−70	−10	2866	5.24***
R Fusiform	26	−70	−8		4.82
R Thalamus	12	−18	8	475	5.10**
L Thalamus	−12	−20	6	371	5.04*
*Conjunction of Similar, Opposite and irrelevant Traits*					
R insula	32	24	0	617	7.62**
SMA	4	16	52	17391	7.55***
	−4	12	52		6.98
L inferior Parietal	−32	−52	46		6.79
L Lingual	−20	−70	−10	2797	5.24***
R Fusiform	26	−70	−8		4.82
R Thalamus	12	−18	8	454	5.10**
L Thalamus	−12	−20	6	340	5.04*

Coordinates refer to the MNI (Montreal Neurological Institute) stereotaxic space. Listed are clusters thresholded at *p* < 0.05, FWE cluster-corrected. SMA = supplementary motor area.

*p < 0.05, **p < 0.01, ***p < 0.001 (FWE cluster-corrected).
