# Corrigendum: The neural representation of competence traits: An fMRI study

**DOI:** 10.1038/srep40972

**Published:** 2017-01-23

**Authors:** Ning Ma, Simin Wang, Quansen Yang, Tingyong Feng, Frank Van Overwalle

Scientific Reports 6: Article number: 3960910.1038/srep39609; published online: 12 20 2016; updated: 01 23 2017

This Article contains a typographical error in the Affiliation of the author Tingyong Feng. The correct affiliation is listed below:

School of Psychology, Southwest University, Chongqing, China.

